# The metabolic change trajectory of skeletal muscle during the life cycle: arteriovenous metabolomics

**DOI:** 10.1093/lifemedi/lnag014

**Published:** 2026-04-27

**Authors:** Siyuan Huang, Yuanping Gu, Guogang Xu, Jing Han, Hao Jia, Yifan Wang, Xiao Chen, Ningning Zhang, Xiumeng Hua, Han Mo, Zhe Sun, Fei Dong, Yuan Chang, Hao Cui, Jiangping Song

**Affiliations:** State Key Laboratory of Cardiovascular Disease, Fuwai Hospital, National Center for Cardiovascular Diseases, Chinese Academy of Medical Sciences and Peking Union Medical College, Beijing 100037, China; Department of Cardiac Surgery, Fuwai Yunnan Hospital, Chinese Academy of Medical Sciences, Affiliated Cardiovascular Hospital of Kunming Medical University, Kunming 650102, China; Health Management Institute, The Second Medical Center & National Clinical Research Center for Geriatric Diseases, Chinese PLA General Hospital, Beijing 100853, China; State Key Laboratory of Cardiovascular Disease, Fuwai Hospital, National Center for Cardiovascular Diseases, Chinese Academy of Medical Sciences and Peking Union Medical College, Beijing 100037, China; State Key Laboratory of Cardiovascular Disease, Fuwai Hospital, National Center for Cardiovascular Diseases, Chinese Academy of Medical Sciences and Peking Union Medical College, Beijing 100037, China; State Key Laboratory of Cardiovascular Disease, Fuwai Hospital, National Center for Cardiovascular Diseases, Chinese Academy of Medical Sciences and Peking Union Medical College, Beijing 100037, China; State Key Laboratory of Cardiovascular Disease, Fuwai Hospital, National Center for Cardiovascular Diseases, Chinese Academy of Medical Sciences and Peking Union Medical College, Beijing 100037, China; State Key Laboratory of Cardiovascular Disease, Fuwai Hospital, National Center for Cardiovascular Diseases, Chinese Academy of Medical Sciences and Peking Union Medical College, Beijing 100037, China; State Key Laboratory of Cardiovascular Disease, Fuwai Hospital, National Center for Cardiovascular Diseases, Chinese Academy of Medical Sciences and Peking Union Medical College, Beijing 100037, China; Department of Cardiac Surgery, Fuwai Hospital, National Center for Cardiovascular Diseases, Chinese Academy of Medical Sciences and Peking Union Medical College, Beijing 100037, China; Shenzhen Key Laboratory of Cardiovascular Disease, Fuwai Hospital Chinese Academy of Medical Sciences, Shenzhen 518057, China; Shenzhen Key Laboratory of Cardiovascular Disease, Fuwai Hospital Chinese Academy of Medical Sciences, Shenzhen 518057, China; Shenzhen Key Laboratory of Cardiovascular Disease, Fuwai Hospital Chinese Academy of Medical Sciences, Shenzhen 518057, China; State Key Laboratory of Cardiovascular Disease, Fuwai Hospital, National Center for Cardiovascular Diseases, Chinese Academy of Medical Sciences and Peking Union Medical College, Beijing 100037, China; Department of Cardiac Surgery, Fuwai Hospital, National Center for Cardiovascular Diseases, Chinese Academy of Medical Sciences and Peking Union Medical College, Beijing 100037, China; State Key Laboratory of Cardiovascular Disease, Fuwai Hospital, National Center for Cardiovascular Diseases, Chinese Academy of Medical Sciences and Peking Union Medical College, Beijing 100037, China; State Key Laboratory of Cardiovascular Disease, Fuwai Hospital, National Center for Cardiovascular Diseases, Chinese Academy of Medical Sciences and Peking Union Medical College, Beijing 100037, China; Department of Cardiac Surgery, Fuwai Yunnan Hospital, Chinese Academy of Medical Sciences, Affiliated Cardiovascular Hospital of Kunming Medical University, Kunming 650102, China; Department of Cardiac Surgery, Fuwai Hospital, National Center for Cardiovascular Diseases, Chinese Academy of Medical Sciences and Peking Union Medical College, Beijing 100037, China; Shenzhen Key Laboratory of Cardiovascular Disease, Fuwai Hospital Chinese Academy of Medical Sciences, Shenzhen 518057, China; Beijing Key Laboratory of Preclinical Research and Evaluation for Cardiovascular Implant Materials, Animal Experimental Centre, Fuwai Hospital, National Centre for Cardiovascular Disease, Chinese Academy of Medical Sciences and Peking Union Medical College, Beijing 100037, China

**Dear Editor,** 

Skeletal muscle accounts for 40% of our body weight and is essential for movement. The aging process has a significant impact on skeletal muscle, which is characterized by a gradual loss of muscle mass and function. This is the primary cause of falls and fractures and the second leading cause of death and injury in elderly individuals [1, 2]. Aging causes increasing metabolic disorder in muscle, including reduced insulin sensitivity, metabolic inflexibility, increased oxidative damage, and mitochondrial dysfunction [3]. Previous research has revealed that fatty acid oxidation, electron transport efficiency, and mitochondrial biogenesis pathways are reduced in muscle tissue of older mice [4]. Metabolomics of human peripheral blood revealed significant perturbations in steroid, amino acid, lipid, and purine metabolism in older adults [5, 6]. Tissue and peripheral venous blood samples were most frequently utilized for metabolomics analysis. The metabolite levels measured, however, are the convergent outcomes of both production and consumption from metabolic processes, which may not indicate changes in metabolic pathway activity [7].

Measuring the arteriovenous (A-V) gradient of circulating metabolites is an effective technique for determining organ-specific metabolite synthesis and consumption [8, 9]. However, limited arterial/deep vein access has restricted A-V gradient use in metabolomics. Here, we analyzed the circulatory A-V metabolome using paired left atrial and femoral venous plasma (Fig. 1A). We enrolled 178 patients receiving transcatheter occlusion of atrial septal defects, including 56 males and 122 females. Participants ranged in age from 14 to 70 (Fig. 1B). A total of 300 known metabolites were quantified using liquid chromatography-mass spectrometry, with 130 metabolites reliably detected in plasma. These metabolites mainly included organic acids and lipids (Fig. 1C). The detected metabolites were predominantly enriched in the amino acid metabolism and biosynthesis pathways (Fig. 1D).

**Figure 1.**
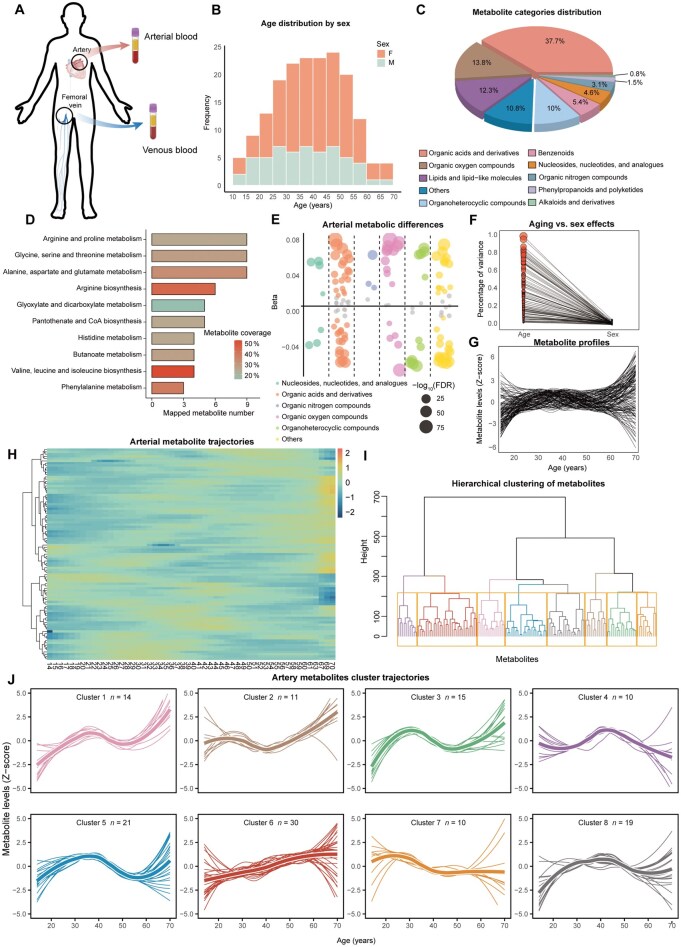
Workflow, metabolite profile, and characteristics of arterial metabolite changes with age.(A) Workflow of the study. Blood samples were collected from the left atrium (A) and femoral vein (V) of participants, followed by plasma preparation for metabolomics analysis. (B) Age and sex distribution of the study population. The frequency of participants (*n* = 178, aged 14 − 70 years) is shown, stratified by sex (male and female). (C) Distribution of metabolite categories. The pie chart shows the proportion of detected metabolites across various functional categories. (D) Metabolic pathway enrichment analysis. The bar chart illustrates the number of mapped metabolites and their coverage in different KEGG pathways. (E) Analysis of arterial metabolite differences. The scatter plot shows the trends of different metabolites with age, categorized by functional groups. The size of the dots represents the significance level (−log_10_(FDR)), with gray dots indicating non-significant metabolites. The *y*-axis represents the effect size of metabolites (beta values). (F) Effects of age and sex on metabolite changes. The line plot shows that the variance explained by age (*R*^2^) in metabolite changes is significantly higher than that explained by sex, indicating that age is the primary driver of metabolic changes. (G) Overall trends of metabolite changes with age. The line plot displays the concentration changes of all metabolites (standardized as *Z*-scores) across the age range of 14 to 70 years, revealing the overall dynamic characteristics of age-related changes. (H) Heatmap of metabolite trajectories. The heatmap illustrates the patterns of metabolite changes with age, with metabolites grouped by hierarchical clustering. The colors represent the standardized metabolite levels (*Z*-scores). (I) Hierarchical clustering of metabolites (based on the WARD.D2 method). The hierarchical clustering divides metabolites into multiple clusters, with each cluster containing metabolites that exhibit similar patterns of age-related changes. (J) Cluster trajectories of metabolites. The line plots show the concentration trends of metabolites in each cluster with age, illustrating the dynamic changes of different metabolite groups. The colors of each cluster correspond to the cluster colors in (I).

Initially, we conducted an analysis and description of the changes in arterial plasma metabolites with advancing age. Logistic regression identified age-correlated metabolites. The typical differentially expressed metabolites (DEMs) based on the minimum *P-*value were labeled in Fig. 1E. Given the greater average longevity of females compared to males, we also evaluated the impact of gender on the metabolome (Fig. 1F). Metabolite expression patterns were heterogeneous across the lifespan, particularly in young and elderly groups (Fig. 1G and 1H). Unsupervised hierarchical clustering identified eight age-related trajectory clusters (10−30 metabolites each) (Fig. 1I). The largest cluster showed linear changes (cluster 6), but others had obviously nonlinear trajectories, such as S-shaped (clusters 1, 3, 5), V-shapedc (cluster 2), and inverted V-shaped (clusters 4, 8). Six of these clusters were enriched in particular biological pathways, demonstrating variations in metabolic processes in human body systems over the lifespan (Table S1). For example, metabolites in blood microparticles increased with age (cluster 6), while metabolites involved in axon guidance and ephrin signaling increased before age 35 and subsequently decreased exponentially from 35 to 50 years old (clusters 1, 5) (Fig. 1J). Most arterial plasma metabolites alter nonlinearly throughout life. The correlation network analysis revealed functional metabolite modules under specific biological conditions, showing chord plots of metabolite correlations in the young group (15−35 years old) and the elderly group (50−70 years old). Specific positive correlations between organic acids and derivatives and organic oxygen compounds were weakened in the elderly group compared to the young group (Fig. S1).

The logistic regression results of age-related venous plasma metabolites were similar to those of arterial metabolites (Fig. S2A). The impact of gender on the metabolome was also comparable (Fig. S2B). The fluctuations of metabolites throughout the lifespan also exhibited patterns akin to arterial metabolomes (Fig. 2A). Unsupervised hierarchical clustering identified 8 age-related trajectory clusters (11−26 metabolites each) (Fig. S2C and S2D). Similar to arterial metabolomes, several distinct nonlinear trajectories, including S-shaped (clusters 1, 3, 5), V-shaped (cluster 6), and inverted V-shaped trajectories (clusters 4, 8), were observed in venous metabolomes. Notably, unlike the arterial metabolome, the venous metabolome lacked a linearly increasing cluster; instead, cluster 2 exhibited exponential growth after age 40. Among the 8 clusters, 6 were enriched in specific biological pathways (Table S2), indicating lifespan variations in lower limb skeletal muscle metabolism. For instance, metabolite levels in blood microparticles continuously increased before the age of 35, gradually decreased between 35  and  50, and then experienced an exponential increase again (clusters 1, 5). In contrast, metabolite levels involved in axon guidance and ephrin signaling remained constant before the age of 35, then gradually rose exponentially (cluster 2) (Fig. 2B). Venous plasma metabolites also displayed predominantly nonlinear changes throughout the lifespan. The results were different from those of the arterial metabolome, with no obvious differences found in metabolite–metabolite correlations between the young and elderly groups (Fig. S2E).

**Figure 2.**
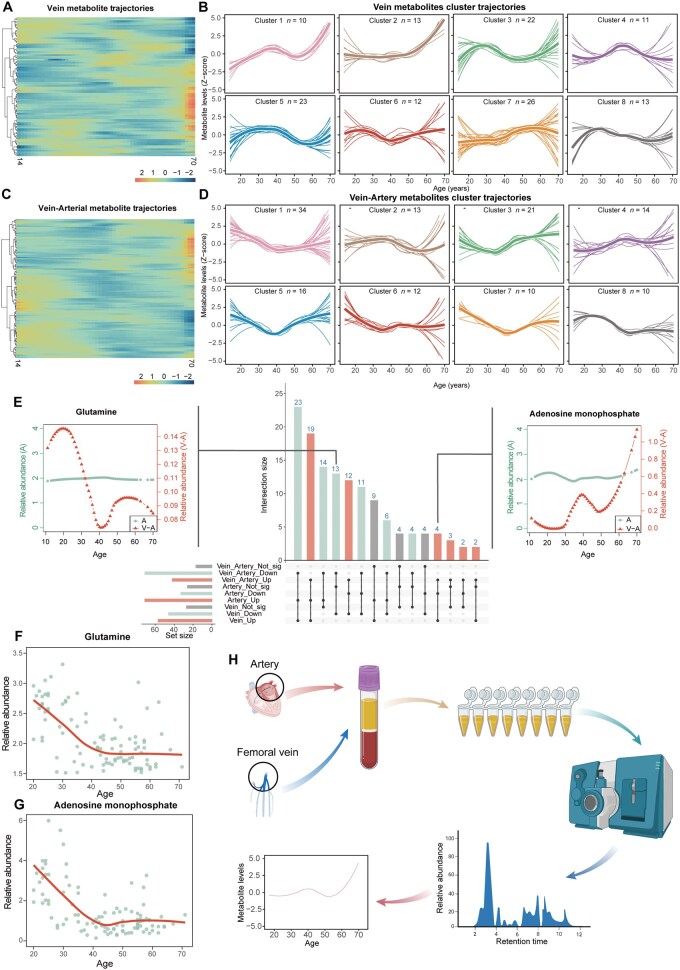
Characteristics and validation of venous, arterial, and venous-arterial metabolite changes with age. (A) Heatmap of venous metabolite trajectories. The heatmap illustrates the patterns of venous metabolite changes with age, with metabolites grouped by hierarchical clustering. The colors represent the standardized metabolite levels (*Z*-scores). (B) Cluster trajectories of venous metabolites. The line plots show the concentration trends of metabolites in each cluster with age, illustrating the dynamic changes of different metabolite groups. The colors of each cluster correspond to the cluster colors in Fig. S2D. (C) Heatmap of venous-arterial metabolite trajectories. The heatmap illustrates the patterns of venous metabolite changes with age, with metabolites grouped by hierarchical clustering. The colors represent the standardized metabolite levels (*Z*-scores). (D) Cluster trajectories of venous-arterial metabolites. The line plots show the concentration trends of metabolites in each cluster with age, illustrating the dynamic changes of different metabolite groups. The colors of each cluster correspond to the cluster colors in Fig. S3D. (E) Left and right subplots: Using glutamine and adenosine monophosphate as examples, this panel illustrates the changes in metabolite levels with age in the arterial group (A values, blue curve) and the venous-arterial group (V-A values, red curve). The black *y*-axis represents A value, while the red *y*-axis represents V-A values. The *x*-axis represents age. Bottom set size bar chart: Shows the number of metabolites in each category, including venous upregulated (Vein Up), venous downregulated (Vein Down), arterial upregulated (Artery Up), arterial downregulated (Artery Down), and venous-arterial differential significance groups (Vein-Artery Up/Down/Not sig).Central upset plot: Displays the intersection relationships and sizes of different metabolite categories. The bar chart’s *y*-axis represents the number of metabolites in each intersection, with the top numbers indicating the intersection size. The horizontal set relationship diagram at the bottom shows the specific category intersections. (F) Validation of glutamine: Demonstrates the age-related changes in glutamine levels in peripheral venous blood from a healthy population. The red smoothing line represents the Loess-fitted curve. (G) Validation of adenosine monophosphate: Illustrates the age-related changes in adenosine monophosphate levels in peripheral venous blood from a healthy population. The red smoothing line represents the Loess-fitted curve. (H) Schematic representation of a workflow for UHPLC-based targeted metabolomics analysis of age-related changes in lower limb arterial and venous metabolites.

To determine the net release and uptake of each metabolite, we calculated the difference in metabolite abundance between venous and arterial plasma. Lower limb utilization of most organic acids and derivatives increased progressively with age (Fig. S3A). However, the difference in metabolite abundance between genders was essentially non-existent (Fig. S3B). Throughout the lifespan, fluctuations in metabolite abundance differences exhibited patterns similar to arterial or venous metabolomes (Fig. 2C). Unsupervised hierarchical clustering revealed eight age-related metabolite trajectory clusters, each containing 10 to 34 metabolites (Fig. S3C and S3D). Unlike arterial or venous metabolomes, there were no linear trajectories in metabolite abundance differences between arteriovenous plasma metabolome. The observed non-linear trajectories were S-shaped (clusters 2, 3, 8), V-shaped (clusters 5, 7), inverted V-shaped (cluster 4), and L-shaped (clusters 1, 6). Notably, the majority of metabolic changes occurred between the ages of 35 and 50. The biggest detected cluster had a continuous drop in metabolite abundance differences before the age of 40 years, followed by a steady trend (cluster 1). Metabolites in this cluster were primarily found in the glycine, serine, threonine, and phenylalanine metabolism pathways (Table S3). Similarly, metabolite abundance disparities in cluster 4 increased until age 40 years, when they stabilized (Fig. 2D). Compared with the young group, the positive metabolite–metabolite correlation was weakened in the elderly group, while the negative correlation was enhanced (Fig. S3E).

Energy metabolism and protein synthesis are central processes to aging. The amount of AMP, a signaling molecule for energy metabolism, varies significantly with age. AMP release increases after age 30, peaks at 40, declines, then spikes exponentially at 50. In contrast, we discovered that glutamine was categorized into cluster 1, indicating an overall decline in metabolite abundance from the ages of 20 to 40 years in venous-arterial plasma. We also found that glutamine did not exhibit substantial age-related changes in arterial plasma, indicating a shift in glutamine metabolic balance over time from release to uptake, eventually reaching a stable state at age 40 years (Fig. 2E). We further investigated age-dependent variations in the level of AMP and glutamine in peripheral venous blood samples from healthy individuals (*n *= 103). The results showed that plasma levels of AMP and glutamine declined steadily before the age of 40 years, then hit a low point around the age of 40 years and remained steady throughout the subsequent life cycle (Fig. 2F and 2G). The results suggested that supplementation with glutamine and AMP, especially after the age of 40 years, may slow down aging by preserving muscle protein synthesis and energy metabolism.

Collectively, our study aimed to investigate the metabolic production and consumption in the skeletal muscle of the lower limb during aging using an arteriovenous sampling approach. The flowchart was shown in Fig. 2H. The metabolic profiles in arterial and venous plasma exhibited similar undulating patterns, showing that metabolic activity in the lower limb muscle is predominantly dependent on arterial input. Metabolic shifts were nonlinear, with prominent changes between ages 35 and 50. After age 40, glutamine release declined while AMP release increased, indicating altered amino acid and energy metabolism with potential therapeutic implications.

## Research limitations

First, it is observational research that restricts the capacity to make causal conclusions. Although the cohort of 178 patients is reasonably sized, a larger sample would enhance the robustness of conclusions concerning age-dependent metabolic alterations. In addition, this study did not incorporate several potential confounding factors, including BMI, lower extremity exercise status, comorbidities, and dietary patterns. Prior research links cellular senescence to glutamine depletion, reversible by supplementation [10]. Our findings demonstrate that venous glutamine content significantly declines with human aging, providing novel insights and evidence regarding glutamine alterations during the aging process. Alterations in arteriovenous glutamine concentration suggest increased glutamine utilization in lower extremity skeletal muscle. Glutamine supports protein synthesis, anti‑inflammatory responses, antioxidant activity, and satellite cell regeneration. These mechanisms may partially elucidate the underlying cause of increased glutamine utilization. However, whether glutamine supplementation can delay aging in human populations remains to be confirmed further in prospective illness cohorts and animal experiments. Furthermore, the metabolomics approach used in this study is based on targeted scanning, which provides restricted coverage, particularly for uncommon metabolites. This may have somewhat impeded our capacity to discover novel molecules and pathways related to metabolic disorders.

## Supplementary Material

lnag014_Supplementary_Data
