# Supplementary material for: The metabolic change trajectory of skeletal muscle during the life cycle: arteriovenous metabolomics
Source: Life Med. 2026 Apr 27;5(3):lnag014. doi: 10.1093/lifemedi/lnag014 (PMC13289604; doi:10.1093/lifemedi/lnag014)
Supplement: lnag014_Supplementary_Data [file lnag014_supplementary_data.docx]

**The metabolic change trajectory of skeletal muscle during the life cycle: arteriovenous** **metabolomics**

Siyuan Huang^1,#^, Yuanping Gu^2,#^, Guogang Xu^3,#^, Jing Han^1^, Hao Jia^1^, Yifan Wang^1^, Xiao Chen^1^, Ningning Zhang^1^, Xiumeng Hua^1,4^, Han Mo^5^, Zhe Sun^5^, Fei Dong^5^, Yuan Chang^1,4^, Hao Cui^1^, Jiangping Song^1,2,4,5,6,*^

^1^State Key Laboratory of Cardiovascular Disease, Fuwai Hospital, National Center for Cardiovascular Diseases, Chinese Academy of Medical Sciences and Peking Union Medical College, Beijing 100037, China

^2^Department of Cardiac Surgery, Fuwai Yunnan Hospital, Chinese Academy of Medical Sciences, Affiliated Cardiovascular Hospital of Kunming Medical University, Kunming 650102, China

^3^Health Management Institute, The Second Medical Center & National Clinical Research Center for Geriatric Diseases, Chinese PLA General Hospital, Beijing 100853, China

^4^Department of Cardiac Surgery, Fuwai Hospital, National Center for Cardiovascular Diseases, Chinese Academy of Medical Sciences and Peking Union Medical College, Beijing 100037, China

^5^Shenzhen Key Laboratory of Cardiovascular Disease, Fuwai Hospital Chinese Academy of Medical Sciences, Shenzhen 518057, China

^6^Beijing Key Laboratory of Preclinical Research and Evaluation for Cardiovascular Implant Materials, Animal Experimental Centre, Fuwai Hospital, National Centre for Cardiovascular Disease, Chinese Academy of Medical Sciences and Peking Union Medical College, Beijing 100037, China

^*^Correspondence: fwsongjiangping@126.com (J.S.)

**Supplemental figures and figure legends**

**
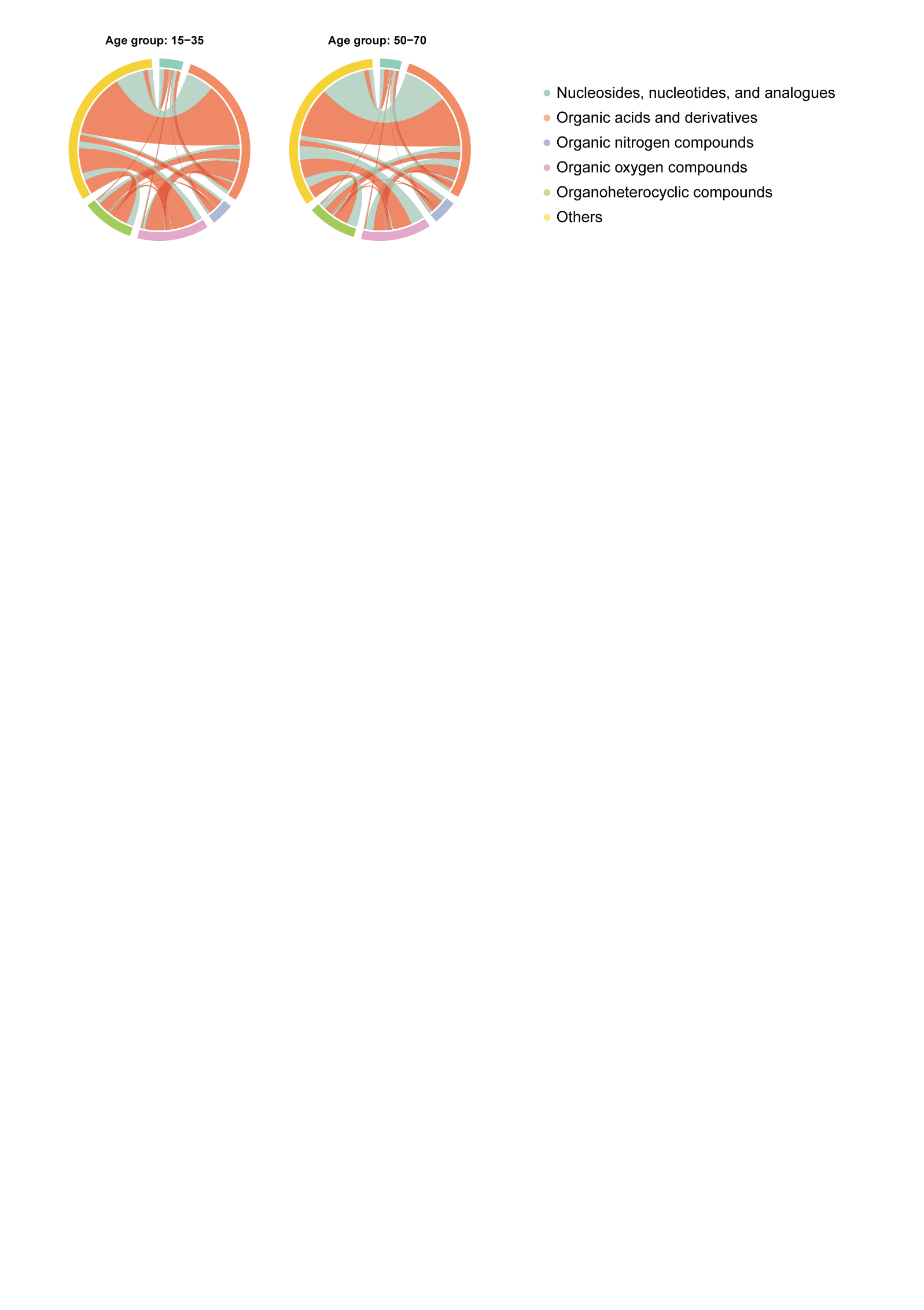
**

**Figure S1. Chord plots of metabolite correlation network analysis between the young and elderly group.**

Artery metabolic functional category association analysis across age groups. The chord diagrams compare the associations and distribution differences of metabolic functional categories between the 15–35 years and 50–70 years age groups. Red chords indicate positive correlations between metabolites, while blue chords indicate negative correlations.

**
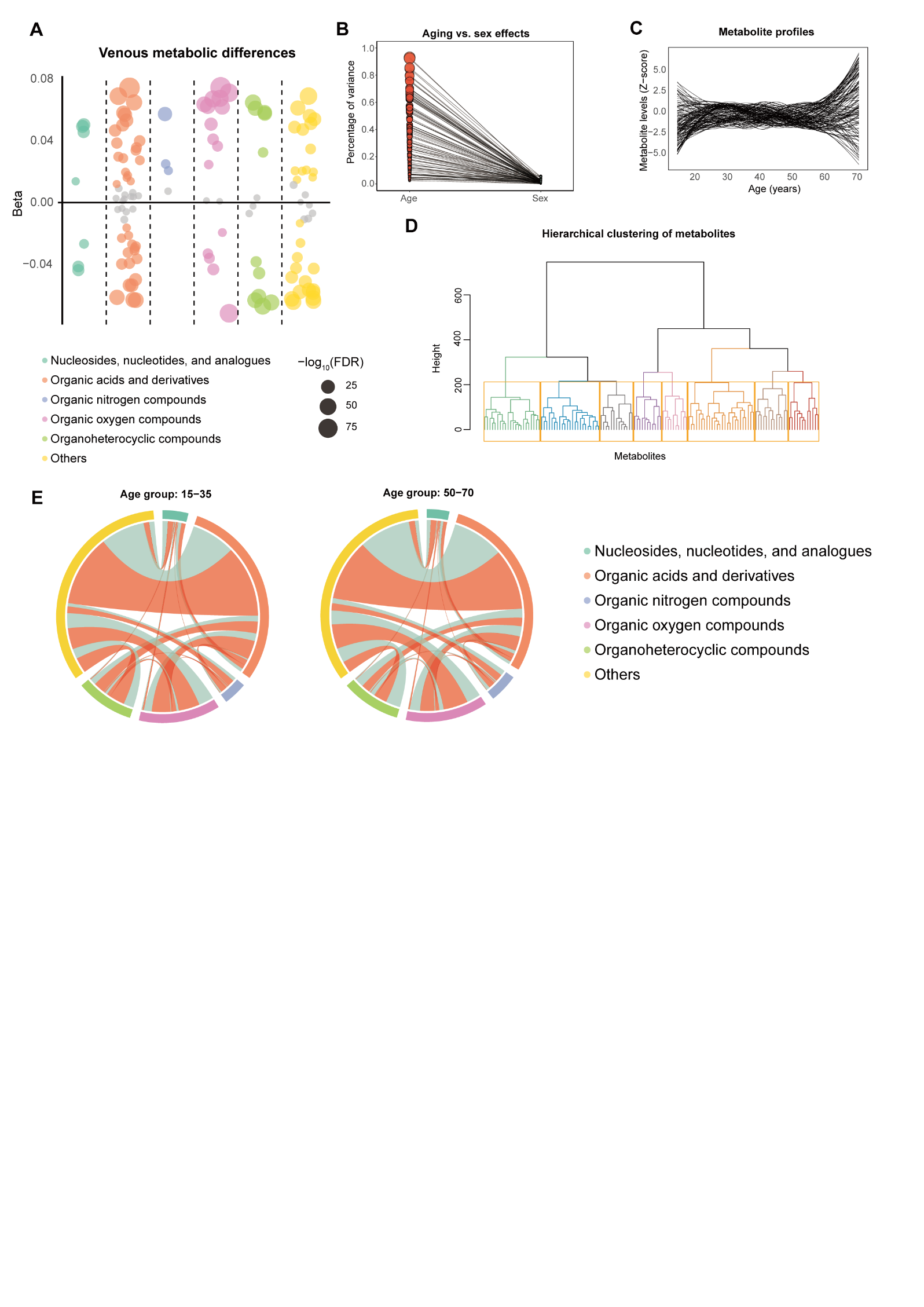
Figure S2. Characteristics of venous metabolite changes with age.**

(A) Venous metabolite differential analysis. The scatter plot shows the trends of different venous metabolites with age, categorized by functional groups. The size of the dots represents the significance level (−log_10_(FDR)), with gray dots indicating non-significant metabolites. The *y*-axis represents the effect size of metabolites (beta values). (B) Effects of age and sex on venous metabolite changes. The line plot shows that the variance explained by age (*R*^2^) in venous metabolite changes is significantly higher than that explained by sex, indicating that age is the main driver of metabolic changes. (C) Overall trends of venous metabolite changes with age. The line plot displays the concentration changes of all venous metabolites (standardized as *Z*-scores) across the age range of 14 to 70 years, revealing the overall dynamic characteristics of age-related changes. (D) Hierarchical clustering of venous metabolites (based on the WARD.D2 method). The hierarchical clustering divides venous metabolites into multiple clusters, with each cluster containing metabolites that exhibit similar patterns of age-related changes. (E) Metabolic functional category association analysis across venous age groups. The chord diagram compares the associations and distribution differences of metabolic functional categories between the 15–35 years and 50–70 years age groups. Red chords indicate positive correlations between metabolites, while blue chords indicate negative correlations.

**
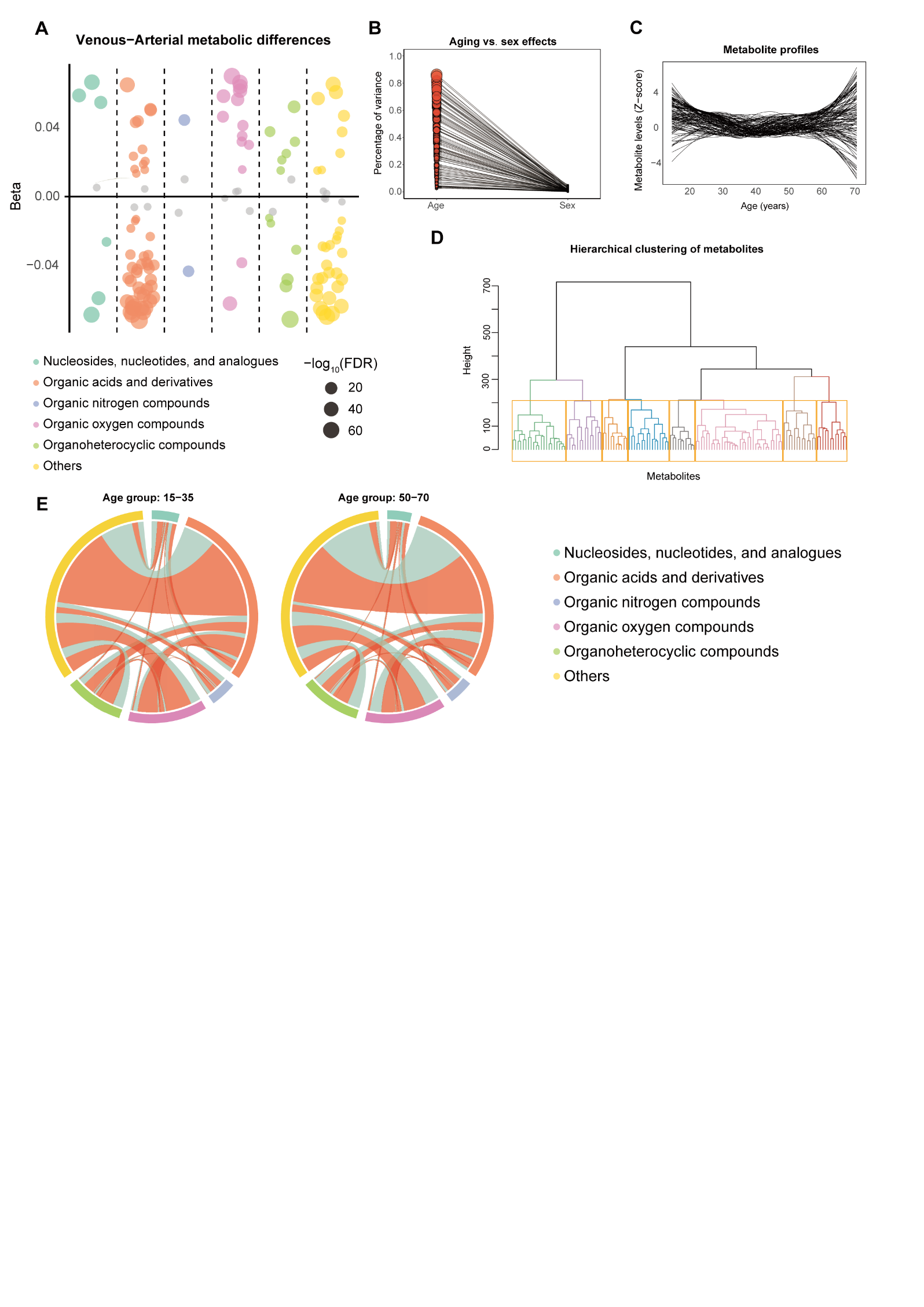
Figure S3. Characteristics of venous-arterial metabolite changes with age.**

(A) Venous-Arterial metabolite differential analysis. The scatter plot shows the trends of different venous-arterial metabolites with age, categorized by functional groups. The size of the dots represents the significance level (−log_10_(FDR)), with gray dots indicating non-significant metabolites. The y-axis represents the effect size of metabolites (beta values). (B) Effects of age and sex on venous-arterial metabolite changes. The line plot shows that the variance explained by age (*R*^2^) in venous-arterial metabolite changes is significantly higher than that explained by sex, indicating that age is the main driver of metabolic changes. (C) Overall trends of venous-arterial metabolite changes with age. The line plot displays the concentration changes of all venous-arterial metabolites (standardized as *Z*-scores) across the age range of 14 to 70 years, revealing the overall dynamic characteristics of age-related changes. (D) Hierarchical clustering of venous-arterial metabolites (based on the WARD.D2 method). The hierarchical clustering divides venous-arterial metabolites into multiple clusters, with each cluster containing metabolites that exhibit similar patterns of age-related changes. (E) Metabolic functional category association analysis across venous-arterial age groups. The chord diagram compares the associations and distribution differences of metabolic functional categories between the 15–35 years and 50–70 years age groups. Red chords indicate positive correlations between metabolites, while blue chords indicate negative correlations.

**Supplemental Tables**

**Supplemental Material Table 1. KEGG pathway analysis results of arterial metabolites in different clusters.**

**Supplemental Material Table 2. KEGG pathway analysis results of venous metabolites in different clusters.**

**Supplemental Material Table 3. KEGG pathway analysis results of venous–arterial metabolites in different clusters.**

**Supplemental Material Table 4. Clinical characteristics of study population.**

| **Table S1. KEGG pathway analysis results of arterial metabolites in different clusters.** | | | | | | | |
| --- | --- | --- | --- | --- | --- | --- | --- |
| **Enrichment Analysis** | **Total** | **Expected** | **Hits** | **Raw *p*** | **Impact** | **FDR** | **Class** |
| Alanine, aspartate and glutamate metabolism | 28 | 0.14 | 2 | 7.99E−03 | 0.05 | 1.60E−02 | 1 |
| Citrate cycle (TCA cycle) | 20 | 0.10 | 2 | 4.10E−03 | 0.10 | 1.64E−02 | 1 |
| Butanoate metabolism | 15 | 0.08 | 2 | 2.29E−03 | 0 | 1.84E−02 | 1 |
| Lipoic acid metabolism | 28 | 0.14 | 2 | 7.99E−03 | 0 | 2.13E−02 | 1 |
| Phenylalanine, tyrosine and tryptophan biosynthesis | 4 | 0.02 | 1 | 2.02E−02 | 0.50 | 2.31E−02 | 1 |
| Tyrosine metabolism | 42 | 0.21 | 2 | 1.76E−02 | 0.14 | 2.34E−02 | 1 |
| Amino sugar and nucleotide sugar metabolism | 42 | 0.21 | 2 | 1.76E−02 | 0 | 2.81E−02 | 1 |
| Phenylalanine metabolism | 8 | 0.04 | 1 | 4.00E−02 | 0 | 4.00E−02 | 1 |
| Taurine and hypotaurine metabolism | 8 | 0.03 | 1 | 3.01E−02 | 0.43 | 3.01E−02 | 2 |
| Primary bile acid biosynthesis | 46 | 0.18 | 2 | 1.16E−02 | 0.02 | 3.49E−02 | 2 |
| Purine metabolism | 70 | 0.27 | 2 | 2.60E−02 | 0.09 | 3.90E−02 | 2 |
| Alanine, aspartate and glutamate metabolism | 28 | 0.20 | 3 | 7.56E−04 | 0.47 | 5.29E−03 | 3 |
| Histidine metabolism | 16 | 0.11 | 2 | 5.05E−03 | 0 | 8.83E−03 | 3 |
| Butanoate metabolism | 15 | 0.10 | 2 | 4.43E−03 | 0.03 | 1.03E−02 | 3 |
| Arginine biosynthesis | 14 | 0.10 | 2 | 3.86E−03 | 0.12 | 1.35E−02 | 3 |
| Neomycin, kanamycin and gentamicin biosynthesis | 2 | 0.01 | 1 | 1.39E−02 | 0 | 1.95E−02 | 3 |
| Arginine and proline metabolism | 36 | 0.25 | 2 | 2.45E−02 | 0.02 | 2.86E−02 | 3 |
| Nitrogen metabolism | 6 | 0.04 | 1 | 4.12E−02 | 0 | 4.12E−02 | 3 |
| Glycine, serine and threonine metabolism | 33 | 0.10 | 2 | 4.09E−03 | 0.21 | 1.23E−02 | 4 |
| Valine, leucine and isoleucine biosynthesis | 8 | 0.03 | 1 | 2.52E−02 | 0 | 3.78E−02 | 4 |
| D-Amino acid metabolism | 15 | 0.05 | 1 | 4.68E−02 | 0 | 4.68E−02 | 4 |
| Glutathione metabolism | 28 | 0.23 | 2 | 2.11E−02 | 0.01 | 2.11E−02 | 5 |
| Alanine, aspartate and glutamate metabolism | 28 | 0.23 | 2 | 2.11E−02 | 0 | 2.63E−02 | 5 |
| Galactose metabolism | 27 | 0.22 | 2 | 1.97E−02 | 0.06 | 3.28E−02 | 5 |
| Glycolysis / Gluconeogenesis | 26 | 0.21 | 2 | 1.83E−02 | 0.01 | 4.57E−02 | 5 |
| Neomycin, kanamycin and gentamicin biosynthesis | 2 | 0.02 | 1 | 1.64E−02 | 0. | 8.22E−02 | 5 |
| Arginine and proline metabolism | 36 | 0.32 | 3 | 3.36E−03 | 0.17 | 5.04E−03 | 6 |
| Lysine degradation | 30 | 0.27 | 3 | 1.97E−03 | 0 | 5.92E−03 | 6 |
| Tryptophan metabolism | 41 | 0.36 | 2 | 4.94E−02 | 0.10 | 4.94E−02 | 6 |
| Purine metabolism | 70 | 0.27 | 3 | 1.53E−03 | 0.03 | 1.53E−03 | 7 |
| Valine, leucine and isoleucine biosynthesis | 8 | 0.07 | 3 | 2.41E−05 | 0 | 1.68E−04 | 8 |
| Glycine, serine and threonine metabolism | 33 | 0.27 | 3 | 2.08E−03 | 0.05 | 7.28E−03 | 8 |
| Valine, leucine and isoleucine degradation | 40 | 0.33 | 3 | 3.64E−03 | 0 | 8.49E−03 | 8 |
| Alanine, aspartate and glutamate metabolism | 28 | 0.23 | 2 | 2.11E−02 | 0.11 | 3.69E−02 | 8 |
| Arginine and proline metabolism | 36 | 0.30 | 2 | 3.38E−02 | 0.04 | 3.95E−02 | 8 |
| Phenylalanine, tyrosine and tryptophan biosynthesis | 4 | 0.03 | 1 | 3.26E−02 | 0.50 | 4.57E−02 | 8 |
| Nitrogen metabolism | 6 | 0.05 | 1 | 4.86E−02 | 0 | 4.86E−02 | 8 |

| **Table S2. KEGG pathway analysis results of venous metabolites in different clusters.** | | | | | | | |
| --- | --- | --- | --- | --- | --- | --- | --- |
| **Enrichment Analysis** | **Total** | **Expected** | **Hits** | **Raw *p*** | **Impact** | **FDR** | **Class** |
| Phenylalanine, tyrosine and tryptophan biosynthesis | 4 | 0.02 | 1 | 1.52E−02 | 0.50 | 2.27E−02 | 1 |
| Tyrosine metabolism | 42 | 0.16 | 2 | 9.73E−03 | 0.14 | 2.92E−02 | 1 |
| Phenylalanine metabolism | 8 | 0.03 | 1 | 3.01E−02 | 0 | 3.01E−02 | 1 |
| Taurine and hypotaurine metabolism | 8 | 0.03 | 1 | 3.01E−02 | 0.43 | 3.01E−02 | 2 |
| Butanoate metabolism | 15 | 0.13 | 3 | 2.39E−04 | 0.03 | 1.67E−03 | 3 |
| Alanine, aspartate and glutamate metabolism | 28 | 0.25 | 3 | 1.61E−03 | 0.47 | 5.63E−03 | 3 |
| Arginine and proline metabolism | 36 | 0.32 | 3 | 3.36E−03 | 0.04 | 7.84E−03 | 3 |
| Histidine metabolism | 16 | 0.14 | 2 | 8.20E−03 | 0 | 9.57E−03 | 3 |
| Nicotinate and nicotinamide metabolism | 15 | 0.13 | 2 | 7.21E−03 | 0.14 | 1.01E−02 | 3 |
| Arginine biosynthesis | 14 | 0.12 | 2 | 6.28E−03 | 0.12 | 1.10E−02 | 3 |
| Purine metabolism | 70 | 0.62 | 3 | 2.15E−02 | 0.04 | 2.15E−02 | 3 |
| Glycine, serine and threonine metabolism | 33 | 0.10 | 2 | 4.09E−03 | 0.21 | 1.23E−02 | 4 |
| Valine, leucine and isoleucine biosynthesis | 8 | 0.03 | 1 | 2.52E−02 | 0 | 3.78E−02 | 4 |
| D-Amino acid metabolism | 15 | 0.05 | 1 | 4.68E−02 | 0 | 4.68E−02 | 4 |
| Alanine, aspartate and glutamate metabolism | 28 | 0.28 | 3 | 2.42E−03 | 0.11 | 1.45E−02 | 5 |
| Arginine biosynthesis | 14 | 0.14 | 2 | 8.20E−03 | 0.06 | 2.46E−02 | 5 |
| Galactose metabolism | 27 | 0.27 | 2 | 2.93E−02 | 0.06 | 3.52E−02 | 5 |
| Phenylalanine, tyrosine and tryptophan biosynthesis | 4 | 0.04 | 1 | 4.01E−02 | 0.50 | 4.01E−02 | 5 |
| Neomycin, kanamycin and gentamicin biosynthesis | 2 | 0.02 | 1 | 2.02E−02 | 0 | 4.04E−02 | 5 |
| Glycolysis / Gluconeogenesis | 26 | 0.26 | 2 | 2.73E−02 | 0.01 | 4.09E−02 | 5 |
| Glycine, serine and threonine metabolism | 33 | 0.13 | 2 | 6.06E−03 | 0 | 1.21E−02 | 6 |
| Purine metabolism | 70 | 0.27 | 2 | 2.60E−02 | 0.01 | 2.60E−02 | 6 |
| Arginine biosynthesis | 14 | 0.12 | 2 | 5.41E−03 | 0.08 | 2.71E−02 | 7 |
| Lysine degradation | 30 | 0.25 | 2 | 2.40E−02 | 0 | 3.00E−02 | 7 |
| Arginine and proline metabolism | 36 | 0.30 | 2 | 3.38E−02 | 0.12 | 3.38E−02 | 7 |
| Lipoic acid metabolism | 28 | 0.23 | 2 | 2.11E−02 | 0 | 3.51E−02 | 7 |
| Neomycin, kanamycin and gentamicin biosynthesis | 2 | 0.02 | 1 | 1.64E−02 | 0 | 4.11E−02 | 7 |
| Valine, leucine and isoleucine biosynthesis | 8 | 0.04 | 2 | 6.23E−04 | 0 | 3.11E−03 | 8 |
| Amino sugar and nucleotide sugar metabolism | 42 | 0.21 | 2 | 1.76E−02 | 0.10 | 1.76E−02 | 8 |
| Glycine, serine and threonine metabolism | 33 | 0.17 | 2 | 1.10E−02 | 0.05 | 1.84E−02 | 8 |
| Valine, leucine and isoleucine degradation | 40 | 0.20 | 2 | 1.60E−02 | 0 | 2.00E−02 | 8 |
| Porphyrin metabolism | 31 | 0.16 | 2 | 9.75E−03 | 0.03 | 2.44E−02 | 8 |

| **Table S3. KEGG pathway analysis results of venous-arterial metabolites in different clusters.** | | | | | | | |
| --- | --- | --- | --- | --- | --- | --- | --- |
| **Enrichment Analysis** | **Total** | **Expected** | **Hits** | **Raw *p*** | **Impact** | **FDR** | **Class** |
| Glycine, serine and threonine metabolism | 33 | 0.42 | 5 | 3.66E−05 | 0.36 | 3.66E−04 | 1 |
| Phenylalanine metabolism | 8 | 0.10 | 3 | 9.43E−05 | 0.36 | 4.72E−04 | 1 |
| Phenylalanine, tyrosine and tryptophan biosynthesis | 4 | 0.05 | 2 | 9.06E−04 | 1.00 | 3.02E−03 | 1 |
| Nitrogen metabolism | 6 | 0.08 | 2 | 2.23E−03 | 0 | 5.58E−03 | 1 |
| Valine, leucine and isoleucine biosynthesis | 8 | 0.10 | 2 | 4.10E−03 | 0 | 8.20E−03 | 1 |
| Glyoxylate and dicarboxylate metabolism | 32 | 0.41 | 3 | 6.87E−03 | 0.04 | 1.15E−02 | 1 |
| Arginine and proline metabolism | 36 | 0.46 | 3 | 9.57E−03 | 0.05 | 1.37E−02 | 1 |
| Arginine biosynthesis | 14 | 0.18 | 2 | 1.27E−02 | 0.12 | 1.59E−02 | 1 |
| Alanine, aspartate and glutamate metabolism | 28 | 0.36 | 2 | 4.75E−02 | 0.31 | 4.75E−02 | 1 |
| Pantothenate and CoA biosynthesis | 20 | 0.11 | 2 | 5.23E−03 | 0.03 | 2.09E-02 | 2 |
| Primary bile acid biosynthesis | 46 | 0.26 | 2 | 2.64E−02 | 0.02 | 3.52E−02 | 2 |
| Pyrimidine metabolism | 39 | 0.22 | 2 | 1.93E−02 | 0.05 | 3.86E−02 | 2 |
| Taurine and hypotaurine metabolism | 8 | 0.05 | 1 | 4.49E−02 | 0.43 | 4.49E−02 | 2 |
| Galactose metabolism | 27 | 0.21 | 3 | 8.93E−04 | 0.06 | 6.25E−03 | 3 |
| Arginine biosynthesis | 14 | 0.11 | 2 | 4.60E−03 | 0 | 1.61E−02 | 3 |
| Fructose and mannose metabolism | 20 | 0.15 | 2 | 9.37E−03 | 0 | 1.64E−02 | 3 |
| Pentose and glucuronate interconversions | 19 | 0.14 | 2 | 8.47E−03 | 0.24 | 1.98E−02 | 3 |
| Alanine, aspartate and glutamate metabolism | 28 | 0.21 | 2 | 1.80E−02 | 0.27 | 2.10E−02 | 3 |
| Neomycin, kanamycin and gentamicin biosynthesis | 2 | 0.02 | 1 | 1.52E−02 | 0 | 2.13E−02 | 3 |
| Amino sugar and nucleotide sugar metabolism | 42 | 0.32 | 2 | 3.87E−02 | 0 | 3.87E−02 | 3 |
| Neomycin, kanamycin and gentamicin biosynthesis | 2 | 0.01 | 1 | 6.34E−03 | 0 | 1.27E−02 | 4 |
| Purine metabolism | 70 | 0.22 | 2 | 1.79E−02 | 0.07 | 1.79E−02 | 4 |
| Glycine, serine and threonine metabolism | 33 | 0.19 | 2 | 1.40E−02 | 0.26 | 1.40E−02 | 5 |
| Arginine and proline metabolism | 36 | 0.16 | 2 | 9.93E−03 | 0 | 9.93E−03 | 6 |
| Alanine, aspartate and glutamate metabolism | 28 | 0.14 | 2 | 7.99E−03 | 0 | 1.60E−02 | 7 |
| Valine, leucine and isoleucine biosynthesis | 8 | 0.04 | 1 | 4.00E−02 | 0 | 4.00E−02 | 7 |
| Arginine and proline metabolism | 36 | 0.14 | 2 | 7.19E−03 | 0.18 | 1.44E−02 | 8 |
| Valine, leucine and isoleucine biosynthesis | 8 | 0.03 | 1 | 3.01E−02 | 0 | 3.01E−02 | 8 |

**Table S4. Clinical characteristics of study population.**

|  | Study population (*n* = 178) |
| --- | --- |
| Age (years) | 40 (30–50) |
| Male, *n* (%) | 56 (31) |
| BMI (kg/m^2^) | 23 (21.1–25.3) |
| Systolic blood pressure (mmHg) | 118 (110–126) |
| Diastolic blood pressure (mmHg) | 74 (69–80) |
| Heart rate (bpm) | 78 (75–82) |
| LVEF (%) | 65 (63–67) |
| Albumin (g/L) | 43.7 (41.4–45.3) |
| hs-CRP (mg/L) | 0.51 (0.27–1.07) |
| Glucose (mmol/L) | 5.03 (4.77–5.42) |
| HbA1c (%) | 5.5 (5.3–5.8) |
| Triglycerides (mmol/L) | 0.91 (0.705–1.32) |
| Cholesterol (mmol/L) | 4.25 (3.69–5) |
| HDL-C (mmol/L) | 1.39 (1.155–1.6) |
| LDL-C (mmol/L) | 2.41 (1.95–2.96) |
| NTproBNP (pg/ml) | 61 (36.3–114.5) |
| Creatine (μmol/L) | 73.4 (65–83) |
| Uric acid (μmol/L) | 304 (247–371) |

Data are presented as median (25th–75th percentiles) counts or *n* (%).

BMI, Body mass index; LVEF, Left ventricular ejection fraction; hs-CRP, High-sensitivity C-reactive protein CRP; HbA1c, Glycosylated hemoglobin Type A1c; HDL-C, high-density lipoprotein cholesterol; LDL-C, low-density lipoprotein cholesterol; NT-proBNP, N-terminal pro-B-type natriuretic peptide.

**METHODS**

**Study population**

All patients were undergoing transcatheter occlusion for treatment of an atrial septal defect without right-to-left or bidirectional shunt. A total of 178 patients were included in this study, ranging in age from 14 to 70 years. The exclusion criteria included prior treatment of percutaneous coronary intervention or coronary artery bypass grafting, hypertension, hyperlipidemia, diabetes, long-term medication, physical impairment, and being combined with major chronic diseases. Venous blood samples for AMP and glutamine quantification were obtained from 103 healthy individuals undergoing medical examination. The study protocol was approved by the Ethics Committee of the Fuwai Hospital of the Chinese Academy of Medical Sciences and Peking Union Medical College (CAMS&PUMC), which was carried out in accordance with the Declaration of Helsinki. All participants provided written informed consent. The detailed information of all patients is in Supplemental Materials Table 4.

**Blood collection from the left atrium and femoral vein**

After local anesthesia, a catheter was placed into the femoral vein, followed by hemostatic sheaths being placed, and the venous samples were collected. Before transcatheter occlusion, arterial samples were collected from the left atrium through the atrial septal defect. Blood samples were collected into EDTA-K2 tubes and immediately placed on ice. Plasma was separated using a 3000 *g* centrifuge at 4°C for 10 minutes and kept at −80°C until analysis.

**Metabolite extraction**

After thawing the plasma samples on ice, 50 μL of plasma was collected and mixed with 0.45 mL of pre-cooling (−40°C) extraction buffer (H_2_O:MeOH:ACN, 20:40:40, *v*/*v*/*v*). The mixture was vortexed for 1 minute before being centrifuged at 18,000 *g* for 30 minutes at 4°C. The supernatant was collected and evaporated using a SpeedVac (Eppendorf, Germany), and the protein pellet was collected for quantification. The pellet was stored at −80°C and redissolved in 100 μL extraction buffer before LC-MS analysis.

**LC–MS/MS analysis**

An established method was used for targeted metabolomic analysis, as described previously[1]. In brief, a UPLC system (Shimadzu Nexera X2 LC-30A) was employed, and the separation was performed on an ACQUITY BEN Amide column (50 × 2.1 mm, 1.7 μm, Waters). A gradient of mobile phase A (H_2_O:ACN, 95:5, *v*/*v*, with 10 mM NH_4_COOH) and mobile phase B (H_2_O:ACN, 5:95, *v*/*v*, with 10 mM NH_4_COOH) was established as follows: 0–1.5 min 95% B; 1.5–6.5 min 95%–70% B; 6.5–10.5 min 70%–30% B; 10.5–13.0 min 30% B; 13.0–13.5 min 30%–95% B; and 13.5–15.0 min 95% B. The flow rate was 0.4 mL/min, the column temperature was 45°C, the injection volume was 15 μL, and the samples were maintained at 4°C in the autosampler. MS was performed with a triple-quadrupole mass spectrometer (QTRAP 6500+, SCIEX) was used with an electrospray ionization (ESI) source in multiple reaction monitoring (MRM) mode. For metabolite identification, the parent (Q1) and product (Q3) ions of MRM transitions of each metabolite were optimized by direct inject analytical standards to the MS, whereas the retention time (RT) of each metabolite was evaluated by inject analytical standards to the LC–MS system. A total of 416 metabolites were monitored with 278 in positive mode and 138 in negative mode. Data acquisition was performed using Analyst 1.7.1 Software (Sciex).

**Data processing**

For targeted metabolomics, chromatogram evaluation and peak area integration were performed using MultiQuant software v.3.0 (SCIEX). Quality control (QC) samples comprised of an equal aliquot of all detected samples were produced and injected once at ten-sample intervals to monitor equipment stability. The metabolites from QC samples with relative standard deviation (RSD) of more than 20% that were excluded from subsequent analysis. Metabolites that were present in at least 80% of samples in both artery and vein groups were kept in the dataset. Missing data were replaced by 1/2th of the minimum value. Finally, 130 metabolites were included in further analysis. The peak area for each metabolite was normalized against the total ion count (TIC), log transformed, and scaled.

**Statistics analysis**

This study employed the LOESS smoothing method to fit the nonlinear trends in metabolites with age and utilized MetaboAnalyst 6.0 (www.metaboanalyst.ca) to classify metabolites into different categories based on their chemical structures. Using a linear regression model (Metabolite ∼ Age + Sex), the regression slope (Beta value) was extracted as an effect size, and the Benjamini-Hochberg method was applied to adjust p-values (FDR < 0.05) to assess the significance of the association between metabolites and age. The coefficient of determination (*R*^2^) was used to evaluate the extent to which age explains the variation in metabolites. Additionally, the Ward.D2 clustering method was used to group the trajectories of metabolite changes with age, revealing distinct patterns of age-related variation. Spearman correlation analysis was performed to evaluate the relationships between metabolites in two age groups (15–35 years and 50–70 years) and to compare the interactions of metabolites from different chemical structure categories. All data processing and analyses were conducted in R (version 4.4.1, 2024-06-14 ucrt).

**Data availability**

The datasets used during this study are accessible from the corresponding author upon reasonable request.

**Research ethics**

This study was approved by the Ethics Committee of Fuwai Hospital (No: 2013−496) and carried out in accordance with the Helsinki Declaration of 1964. The individuals who took part in this study provided written informed consent

**Reference**

[1] Wang W, Cui H, Ran G, Du C, Chen X, Dong S et al: Plasma metabolic profiling of patients with tetralogy of fallot. Clin Chim Acta 2023, 548:1175
